# The immune landscape of esophageal cancer

**DOI:** 10.1186/s40880-019-0427-z

**Published:** 2019-11-26

**Authors:** Tu-Xiong Huang, Li Fu

**Affiliations:** Guangdong Provincial Key Laboratory of Regional Immunity and Diseases, Department of Pathology and Shenzhen International Cancer Center, Shenzhen University Health Science Center, Shenzhen, 518060 Guangdong P. R. China

**Keywords:** Esophageal cancer, Immune landscape, Prognosis, Immunotherapy

## Abstract

Esophageal cancer (EC) seriously threatens human health, and a promising new avenue for EC treatment involves cancer immunotherapy. To improve the efficacy of EC immunotherapy and to develop novel strategies for EC prognosis prediction or clinical treatment, understanding the immune landscapes in EC is required. EC cells harbor abundant tumor antigens, including tumor-associated antigens and neoantigens, which have the ability to initiate dendritic cell-mediated tumor-killing cytotoxic T lymphocytes in the early stage of cancer development. As EC cells battle the immune system, they obtain an ability to suppress antitumor immunity through immune checkpoints, secreted factors, and negative regulatory immune cells. Cancer-associated fibroblasts also contribute to the immune evasion of EC cells. Some factors of the immune landscape in EC tumor microenvironment are associated with cancer development, patient survival, or treatment response. Based on the immune landscape, peptide vaccines, adoptive T cell therapy, and immune checkpoint blockade can be used for EC immunotherapy. Combined strategies are required for better clinical outcome in EC. This review provides directions to design novel and effective strategies for prognosis prediction and immunotherapy in EC.

## Introduction

Esophageal cancer (EC) is the eighth most common cancer type and the sixth leading cause of cancer death worldwide [1]. It mainly includes esophageal squamous cell carcinoma (ESCC) and esophageal adenocarcinoma (EAC) [2]. ESCC makes up most EC cases worldwide and predominates in Eastn Asia and Africa, while EAC is more prevalent in many developed countries [2]. Standard therapy for EC is limited to surgical or endoscopic resection and chemoradiotherapy [2]. Approximately half of EC patients have distant metastases, and these patients are managed primarily with chemotherapy regimens, such as the combination of 5-fluorouracil, cisplatin, and taxanes [3]. However, EC is inherently resistant to chemotherapy as a result of its heterogeneity. Despite recent advances in the treatment of metastatic EC with the addition of targeted therapy to chemotherapy regimens, the prognosis of EC remains relatively poor, with an approximately 15%–25% 5-year survival rate [4]. A promising new avenue for EC treatment involves cancer immunotherapy, and different preclinical or clinical studies of EC immunotherapy, including tumor vaccination, immune checkpoint inhibition, and adoptive T-cell therapy, have been ongoing in recent years [5]. However, EC immunotherapies always lead to mixed results, which are partially caused by the absence of reliable markers that are predictive of treatment response. The combined positive score (CPS) and tumor proportion score (TPS), two immunohistochemical assessments that define programmed death-ligand 1 (PD-L1) expression in cancers, have been used to help identify patients who are likely to respond to programmed cell death protein 1 (PD-1)/PD-L1 blockade [5]. Despite these scoring systems, it remains difficult to accurately predict the response of EC patients to anti-PD-1 antibodies. For example, responses to treatment with anti-PD-1 antibodies can be observed in PD-L1-negative tumors [6]. Moreover, although pembrolizumab is approved by the U.S. Food and Drug Administration (FDA) to treat patients who have progressed after second-line therapies and whose tumors are PD-L1 positive, this anti-PD-1 antibody failed to improve the treatment efficacy compared with paclitaxel as the second-line therapy for patients with advanced PD-L1-positive gastroesophageal cancer [7]. These results suggest that the immune microenvironment in EC may be intractable and that various factors may be implicated in the regulation of antitumor immunity or in the intervention of immunotherapy in EC. Therefore, studies on the initiation and regulation of antitumor immunity in EC are critical to design proper strategies for EC immunotherapy and for the prediction of treatment response.

Recently, the idea of the EC immune microenvironment regulating antitumor immunity has attracted increasing research interest [5, 8]. However, how the tumor microenvironment (TME) regulates antitumor immunity in EC remains unclear, and reviews systematically describing the initiation and regulation of antitumor immunity in EC are scant. In this review, we focus on the immune landscape (including the factors implicated in the initiation or regulation of antitumor immunity and their relationship with cancer prognosis) of EC and the immunotherapy strategies based on these landscapes.

## Initiation of antitumor immunity in EC

EC cells were considered to have high immunogenicity and were able to induce antitumor immunity in the early stage of EC development [9]. Specific tumor antigens, including tumor-associated antigens (TAAs) and tumor-specific antigens, are essential for cancer cells to initiate antitumor immune responses [10]. Cancer-testis antigens (CTAs), which include melanoma-associated antigen-A (MAGE-A), New York esophageal squamous cell carcinoma 1 (NY-ESO-1), Cancer-testis antigen 2 (CTAG2; also known as LAGE1), and TTK protein kinase (TTK), are the best-studied TAAs and are always highly expressed in EC, especially in ESCC. More importantly, the antitumor immunity or antibody response induced by MAGE-A [11] and NY-ESO-1 [12, 13] can be detected in the tumor samples from ESCC patients. Approximately 2.54% of peripheral blood mononuclear cells (PBMCs) from ESCC patients can be detected as MAGE-A3-specific CD8^+^ T cells, and their responses to MAGE-A3 peptide in vitro were also detectable [11]. The NY-ESO-1 autoantibody in the serum can be detected with an OD higher than 0.165 in 32% of ESCC patients [13]. TTK is capable of eliciting potent and specific cytotoxic T lymphocytes (CTLs) in vitro against EC cells expressing TTK [14]. To date, the antitumor immunity induced by CTAG2 has not been reported in EC. It is worth noting that the expression of CTAs may be different between ESCC and EAC. For example, MAGE-A is expressed in most primary ESCCs (> 50%) and all metastatic lymph nodes but in only a small proportion of EACs (~ 15%) [15, 16]. EC cells always harbor a large number of genetic mutations, which generate specific neoantigens [9]. Tumor neoantigens have been identified in EC cell lines [17] and EC tissues [18], and EC-derived neoantigens have been shown to induce specific CTLs [17]. CTL responses against tumors is the key mechanism of killing tumors by the immune system and are always induced by recognition of tumor antigens on antigen-presenting cells (APCs). As the most well studied APCs in antitumor immunity, dendritic cells (DCs) ingest tumor cells and their antigens and then present the antigens to CD8^+^ T cells, thus priming tumor-specific CTLs that are capable of killing tumor cells [19]. Chen et al. [11] detected MAGE-A3-specific CD8^+^ T cells in PBMCs and tumor-infiltrating lymphocytes (TILs) from ESCC patients and showed that these MAGE-A3-specific CD8^+^ T cells were able to respond to MAGE-A3-loaded DCs and kill human leukocyte antigen 2 (HLA-2)^+^MAGE-A3^+^ tumor cells but not HLA-2^−^MAGE-A3^+^ or MAGE-A3^−^ tumor cells, suggesting that DC-mediated, major histocompatibility complex class 1 (MHC-I)-restricted, tumor-specific CTLs can be induced in EC patients. Type 1 T helper (Th1) cells [20, 21] and natural killer (NK) cells [22, 23], two other types of immune cells involved in immune surveillance; have also been found to play roles in antitumor immunity in EC. A B-cell response is also found in EC and can serve as a predictive marker for EC [13, 24], but the role of the B-cell response in initiating or assisting immune surveillance against EC cells remains unclear.

The immune response against EC cells plays a critical role in preventing or controlling the development of EC in early stages. However, as described below, while EC cells battle with the immune system, mutations and/or other alterations in EC cells may endow them with the ability of immune evasion.

## Regulation of antitumor immunity in EC

EC cells have the ability to inhibit the antitumor immunity responsible for killing tumor cells in various ways, including the down-regulation of tumor antigens, MHC molecules or molecules needed for antigen processing, the expression of inhibitory cell surface proteins (also called immune checkpoints), or the secretion of immunosuppressive proteins. Here, we focus on the regulation of antitumor immunity in the esophageal TME. The factors involved in the regulation of antitumor immunity by the TME mainly include immune checkpoints and secreted immunosuppressive proteins derived from cancer cells or stromal cells.

### Immune checkpoints in tumor cells or immune cells

After activation, T cells always express enhanced inhibitory receptors, such as cytotoxic T lymphocyte-associated protein 4 (CTLA-4) and PD-1, for the regulation of immune balance and avoiding excessive immune response [10, 25]. Cancer cells elicit the inhibitory signaling of effector T cells for immune evasion by overexpressing inhibitory molecules or by inducing immune cells to express inhibitory proteins [25]. The expression of cell surface inhibitory molecules, such as PD-L1/2 and VISTA [9, 26, 27], and inhibitory receptors, such as PD-1, CTLA-4, T-cell immunoglobulin and mucin-domain containing-3 (TIM-3), and lymphocyte-activation gene 3 (LAG-3) [28–31], can be detected commonly in EC. It is worth noting that CTLA-4 is generally considered as an inhibitory receptor on immune cells; however, the expression of CTLA-4 in EC is not limited to tumor-infiltrating immune cells (TIICs) but also found in tumor cells [28]. The function of tumor cell-derived CTLA-4 remains unclear. A non-membrane inhibitory molecule, indoleamine 2,3-dioxygenase 1 (IDO1), has recently attracted intensive research interest in the field of EC and can be detected in approximately 20% of ECs [32]. An increased IDO1 level was associated with decreased CD8^+^ T cells, suggesting the immune-suppressive functions of IDO1 in EC [32]. Similar to that of tumor antigens, the expression of immune checkpoints may also vary between ESCC and EAC. Both PD-L1 and PD-L2 are highly expressed in a high proportion of ESCC patients (> 40% in most studies) [9]. Unlike ESCC, EAC seems to preferentially express PD-L2 over PD-L1. Moreover, PD-L1 is expressed in both tumor cells and TIICs in ESCC, but there is significantly preferential expression in TIICs rather than in tumor cells in EAC [26].

### Tumor- or stroma-derived secretome for immune suppression

The secreted chemokines, cytokines, and growth factors derived from cancers always play critical roles in promoting cancer progression in multiple ways, including the inhibition of antitumor immunity. Among these secreted proteins, transforming growth factor-β (TGF-β) is a well-studied tumor cell-derived growth factor involved in the regulation of the antitumor immune response, impairing the cytotoxicity of effector T cells or NK cells and up-regulating the immune checkpoints of regulatory immune cells, such as tumor-associated macrophages (TAMs), myeloid-derived suppressor cells (MDSCs), and suppressive regulatory T cells (Tregs) [33, 34]. The overexpression or hyperactivation of TGF-β can be detected in a high proportion of EC patients [35, 36], and more importantly, blockade of TGF-β enhances the efficiency of PD-L1/PD-1 inhibition, inducing MAGE-A3^+^ specific CD8^+^ T-cell response in ESCC [11]. Increased levels of interleukin-10 (IL-10) were detected and shown to positively correlate with Treg density in ESCC [37]. Treg cell-derived IL-10 can promote the exhaustion of CD8^+^ TILs and thus limit effective antitumor immunity [38]. As another well-known immunosuppressive secreted factor in the TME, interleukin-6 (IL-6) is also highly expressed in some EC cases [39], especially in cancer-associated fibroblasts (CAFs) from EC patients, including both ESCC and EAC patients [40, 41]. IL-6/signal transducer and activator of transcription 3 (STAT3) signaling has been shown to attenuate antitumor immunity by inhibiting DC maturation [42]. More interestingly, increased IL-6 secretion from CAFs is associated with their immunosuppressive phenotype in EC [40]. Other secreted proteins involved in the regulation of antitumor immunity in the TME, such as vascular endothelial growth factor (VEGF), interleukin-8 (IL-8), C–C motif chemokine ligand 2 (CCL2), and C–C motif ligand 5 (CCL5), were also found in EC [40, 43, 44]. In addition to soluble secreted proteins, exosomes derived from tumor cells or stromal cells also play critical roles in the inhibition of antitumor immunity [45]. It has been shown that EC-derived exosomes have the ability to induce regulatory B cells, which produce TGF-β and thus suppress the proliferation and activities of CD8^+^ T cells [46].

### Cancer stromal cells for immune regulation

Cancer cells can inhibit immune surveillance directly by suppressing the initiation of tumor-killing immune response or indirectly by stimulating the activation of regulatory immune cells, such as Tregs, Th17 cells, M2-like TAMs, and MDSCs. High infiltration of Tregs [47], M2-like TAMs [48, 49], and MDSCs [50, 51] can be detected in a high proportion of ESCCs. In EC, Tregs can be recruited by tumor- or stroma-derived chemokines, such as C–C motif ligand 20 (CCL20), and activated by B7/CTLA-4 signaling [52, 53]. After activation, Tregs suppress DC and T cell functions through the secretion of immune-suppressive cytokines, such as IL-10 and TGF-β [53]. M2-like TAMs may play roles in the negative regulation of the antitumor immune response in EC by elevating PD-L1 expression in tumor cells [48] or by recruiting Tregs to the TME through secretion of C–C motif ligand 17 (CCL17) and C–C motif ligand 22 (CCL22) [54]. The activation of MDSCs in EC is regulated by IL-6 or other signaling pathways mediated by aldehyde dehydrogenase 1 (ALDH1) [50, 55]. MDSCs are heterogeneous, and CD38 serves as a marker for MDSCs with increased immunosuppressive capacity in EC [56]. As another T cell subset involved in the regulation of antitumor immunity, Th17 cells can be increased in the peripheral blood or tumor tissues of EC patients compared with the numbers in healthy donors [57]. Interleukin-17A (IL-17A), a Th17 cell-secreted inflammatory cytokine, has been shown to have conflicting roles in regulating tumor development of EC. In one study, IL-17A promoted the invasiveness of EAC cells [58], whereas in another study, it played a protective role in human ESCC by enhancing the cytotoxic effects of NK cells, killing tumor cells, and activating CD1a^+^ DCs in tumors [59]. Therefore, a deeper understanding of the role of Th17 cells in the regulation of antitumor immunity in EC is required in the future.

In addition to suppressive immune cells, CAFs, another type of stromal cell, have also been linked to the negative regulation of antitumor immunity in various cancers, including breast cancer, lung cancer, colon cancer, and pancreatic ductal adenocarcinomas (PDACs) [60]. Evidence for CAF-regulated immune suppression in EC was also found. It has been shown that CAFs with fibroblast activation protein (FAP) expression in EC have the ability to secrete IL-6 and CCL2, which have been considered to be related to the negative regulation of antitumor immunity and thus promote the generation of an immune-suppressive TME by inducing M2 polarization of macrophage-like cells [40, 41]. CCL2 derived from FAP^+^ CAFs was also able to promote the infiltration of MDSCs [61]. Moreover, the hyaluronan synthesis in CAFs modulated by ESCC cells was capable of promoting adhesion of CD4^+^ but not CD8^+^ T cells to xenografted tumor tissues, affecting the tumor immune response.

## Prognostic values of immune landscape in EC

The development of cancer in an individual patient depends on both the tumorigenic activities of tumor cells, such as their growth, metastasis, and therapy resistance abilities, and the characteristics of the TME immune landscape, which include the factors of the tumor-killing immune response and the regulators suppressing antitumor immunity. The antitumor immune landscape in the TME may vary between different individual patients. The prognostic values of some components from the EC immune landscape have been investigated recently (Table 1).

**Table 1:** The prognostic values of immune landscape markers in EC

Biomarker	Prognostic value					
	Clinical survival	Tumor stage	Differentiation grade	Metastasis	Response to chemotherapy	Response to immunotherapy
MAGE-A11	− [63]	N/A	N/A	+ [63]	N/A	N/A
NY-ESO-1	N/A	N/A	N/A	N/A	N/A	Vaccination:− [24]
TMB	N/A	N/A	N/A	N/A	N/A	PD-1 inhibition: + [18]
MANA	N/A	N/A	N/A	N/A	N/A	PD-1 inhibition: + [18]
PD-L1	N/A	N/A	N/A	N/A	N/A	PD-1 inhibition: + [18]
PD-1	N/A	+ [67]	N/A	+ [67]	N/A	N/A
CTLA-4	− [28]	N/A	N/A	N/A	N/A	N/A
IDO1	− [32, 70]	N/A	N/A	N/A	− [68]	N/A
PD-L1 + IDO1	− [68, 69]	N/A	N/A	N/A	− [69]	N/A
VISTA	+ [27]	N/A	N/A	N/A	N/A	N/A
TGF-β	N/A	N/A	N/A	N/A	− [72]	N/A
TGF-β + IL-10	N/A	+ [71]	N/A	N/A	N/A	N/A
IL-6	N/A	N/A	N/A	N/A	− [50]	N/A
CD80 or CD86	N/A	− [71, 73]	− [71, 73]	N/A	N/A	N/A
CD1a^+^ cells	N/A	N/A	− [73]	N/A	N/A	N/A
CD8^+^ TILs	+ [75]	N/A	N/A	− [75]	+ [75]	N/A
CD8^+^/Foxp3^+^ ratio	+ [29]	N/A	N/A	N/A	N/A	N/A
CCL4^high^CCL20^low^	+ [52]	N/A	N/A	N/A	N/A	N/A
M2-like TAMs	− [48, 49]	N/A	N/A	N/A	N/A	N/A
MDSCs	N/A	+ [50, 51]	N/A	N/A	− [50]	N/A

+, positively correlated; −, negatively correlated; *N/A* not available

*EC* esophageal cancer, *MAGE-A11* melanoma-associated antigen A11, *NY-ESO-1* New York esophageal squamous cell carcinoma 1, *TMB* tumor mutation burden, *MANA* mutation-associated neoantigen, *PD-L1* programmed death-ligand 1, *PD-1* programmed cell death protein 1, *CTLA-4* cytotoxic T lymphocyte-associated protein 4, *IDO1* indoleamine 2,3-dioxygenase 1, *TGF-β* transforming growth factor-β, *IL-10* interleukin-10, *IL-6* interleukin-6, *TILs* tumor-infiltrating lymphocytes, *TAM* tumor-associated macrophage, *MDSC* myeloid-derived suppressor cell

### Tumor antigens and relative markers

The TAAs in EC mainly include MAGE-A, NY-ESO-1, CTAG2, and TTK. None of them showed a significant association with disease progression or prognosis in patients with EC [15, 16, 62]. One of the reasons for TAAs not associating with EC patient prognosis may be the dual roles of these TAAs. On the one hand, TAAs could serve as tumor antigens and initiate an immune response to kill tumor cells that express them [11–13], but on the other hand, TAAs have the ability to promote tumor development as oncogenic proteins [63, 64]. However, some subtypes of MAGE-A, such as MAGE-A11, was shown to be associated with distant lymph node metastasis and poor prognosis in ESCC patients [63]. Moreover, NY-ESO-1 expression and immune response are associated with an immuno-suppressive TME and poor prognosis in MAGE-A4-vaccinated patients with ESCC [24, 64]. It is worth noting that, while initiating the immune response against NY-ESO-1^+^ tumor cells as a tumor antigen [13], NY-ESO-1 is able to regulate an immuno-suppressive TME by inducing IDO1 production and Tregs [64]. These studies suggest that NY-ESO-1 could be used as a poor prognosis marker for vaccination therapy in EC patients. Tumor-specific neoantigens also contribute to the initiation of antitumor immunity. Tumor mutation burden (TMB) and microsatellite instability (MSI), which are related to the generation of neoantigens, have been used to predict the response to PD-L1/PD-1 blockade in various tumors [65]. While MSI is rarely found in EC [5], TMB and the mutation-associated neoantigen (MANA) count have been shown to be associated with better therapy response to anti-PD-1 antibodies in ESCC patients [18].

### Genetic alterations for the regulation of antitumor immunity

Many studies have investigated the prognostic value of immune checkpoints in EC. In studies with a relatively small series of patients, PD-L1/2 expression in ESCC was associated with poor prognosis [9]. However, in other studies with larger series of ESCC patients, the high expression of PD-L1 was associated with a well-differentiated disease status, early tumor stage, and increased survival benefits [29, 66]. These conflicting results may have been caused by different patient accounts, different preoperative treatments for patients, different methods or principles for PD-L1/2 detection in these studies, and the complex interplay of the TME and cancer treatments. The prognostic values of PD-L1/2 in EAC remain unclear [9, 26]. However, the expression of their receptor, PD-1, on TILs and cancer cells is associated with tumor stage and lymph node metastasis in EAC [67]. PD-L1 expression is also used as a biomarker for predicting patient response to PD-L1/PD-1 blockade in EC. Huang et al. [18] showed that an objective response to PD-L1/PD-1 blockade was more common in patients with PD-L1-positive ESCC than in those with PD-L1-negative ESCC. However, the difference was not significant. To the best of our knowledge, only one study has investigated the prognostic value of CTLA-4 in EC patients. In this study, CTLA-4 expression in either cancer cells or TIICs was associated with shortened overall survival (OS) in ESCC patients, and the OS of patients with CTLA-4-positive epithelial cells was similar to that of patients with CTLA-4-positive TIICs. Interestingly, the co-expression of tumor cell-derived CTLA-4 and TIIC-derived CTLA-4 can predict the outcomes of ESCC patients more accurately than each marker alone [28]. IDO1 expression is associated with decreased OS, poor pathologic response, and increased recurrence in both ESCCs and EACs [32, 68, 69]. Consistently, IDO1 promoter hypomethylation, which results in the up-regulation of IDO1, is also associated with poor prognosis in EC patients [70]. Moreover, the co-expression of IDO1 and PD-L1 is better for the prediction of EC patient outcomes than either alone [68, 69]. Unlike CTLA-4 and IDO1, another immune checkpoint molecule, VISTA, may emerge as a positive prognostic marker for EC patients, at least for EAC patients. Loeser et al. [27] showed that the expression of VISTA was associated with prolonged OS in EAC patients with stage pT1/2 tumor. As secreted regulators in the TME, TGF-β and IL-10 are associated with the stage of EC, having higher expression in stage III or IV tumors than in stage I or II tumors [71]. Moreover, TGF-β expression is associated with poor therapeutic response and prognosis in patients with EAC [72]. Additionally, the serum IL-10 level was higher in synchronous ESCC than in nonsynchronous ESCC, which always leads to a poor prognosis [37]. Another secreted immune regulator, IL-6, was shown to be associated with a poor therapeutic response in ESCC [50]. The immune microenvironment in EC is characterized by a lack of cytokines and growth factors involved in tumor-killing immune responses, such as interferon-γ(IFN-γ) and granzyme B (GramB), and by high expression of those cytokines and growth factors involved in immune suppression, such as TGF-β, VEGF, IL-10, and IL-8 [35, 43]. Therefore, a combination of multiple markers, including immune-stimulating and immune-suppressive secreted factors, should be developed in the future for better prediction of prognosis in EC.

### Tumor-infiltrating immune cells

The biological activities of immune cells infiltrated into the TME determine the effects of the antitumor immune response. DCs play key roles in the tumor-antigen presentation and the priming of effector T cells, and CD80 and CD86 are two markers of DC maturation and are critical for the activation of costimulatory signaling during DC priming of CD8^+^ T cells [19]. The expression of CD80 and CD86 in EC tissues and regional lymph nodes was significantly down-regulated compared with that in normal esophageal tissues, was negatively associated with tumor stage, was positively associated with tumor differentiation status, and was not associated with clinical survival or lymph node metastasis [71, 73]. Moreover, the number of CD1a^+^ DCs in tumors was also associated with the level of pathologic differentiation (grades 1–2 had higher numbers than grade 3) in EC tumors [73]. Since EC cells have the ability to induce inhibitory DCs expressing IDO1 and/or PD-L1 [5, 32] and DCs with high expression of IDO1 or PD-L1 suppress the antitumor immune response [5, 74], a comprehensive consideration of the infiltration of DCs with different phenotypes, including IDO1^high^B7^low^ and IDO1^low^B7^high^ DCs, would improve the prognostic values of DCs in EC patients. The density and activity of TILs are the key factors determining the effect of the antitumor immune response [19] and thus could be used as prognostic markers for EC. The increase in the number of CD8^+^ TILs is associated with prolonged survival in EC patients, a better pathologic response to neoadjuvant chemotherapy, and a lower rate of lymph node metastasis. Increased CD4^+^ TILs were associated with significant local regression of EC [32, 75]. The combined evaluation of PD-L1 expression in tumors and the degree of activation of TILs has been used to predict the response to PD-L1/PD-1 blockade in various cancers [76]. In EC, increased CD8^+^ TILs are always detected in PD-L1-positive tumors, whereas the levels of cytotoxic T cells are low in PD-L1-negative ones [5], suggesting that enhanced PD-L1 expression in tumors may be caused by increased TILs. Therefore, the prognostic value of CD8^+^ TILs may be similar to that of tumoral PD-L1 expression or to that of both parameters in EC patients receiving PD-L1/PD-1-blocking therapy. Notably, CD4^+^ TILs include Tregs, which suppress the tumor-killing activities of cytotoxic CD8^+^ TILs. Therefore, the comprehensive consideration of tumor-infiltrating CTLs and Tregs would improve the prognostic values of TILs in EC patients [29]. Moreover, increased M2-like TAMs were associated with significantly shorter OS in EC patients [48, 49], and elevated levels of MDSCs were associated with advanced disease stage and poor prognosis in EC patients [50, 51].

### Combined prognosis prediction

A single TME-derived factor is insufficient for accurate cancer prognostic prediction. Thus, comprehensive strategies using multiple factors are required to improve the prognostic value of the immune landscape in EC. The expression of IDO1 was associated with that of PD-L1, and patients with co-expression of IDO1 and PD-L1 had significantly lower therapeutic response and higher recurrence rate than those with either one or none expression in ESCC [68, 69]. Zhou et al. [68] reported that the pathologic complete response (pCR) rates of IDO^+^PD-L1^+^, IDO^+^PD-L1^−^ or IDO^−^PD-L1^+^, and IDO^−^PD-L1^−^ ESCC patients were 21.4%, 34.5%, and 57.3%, respectively (*P* = 0.001). The 3-year recurrence rates for these three groups were 60.0%, 29.8%, and 14.2%, respectively (*P* < 0.001). Except for the combination of different immune checkpoints, the combination of different types of TIICs was also used for EC prognosis prediction [29, 52]. The CD8^+^/Foxp3^+^ ratio was positively correlated with OS in ESCC patients [29], although the infiltrating Tregs alone is not a good marker for the prediction of survival in ESCC patients [77]. Consistently, CCL4 and CCL20, which recruit CTLs and Tregs, respectively, have been considered as strong reciprocal predictive markers for the survival of ESCC patients [52]. CCL4^high^CCL20^low^ ESCC patients have higher 5-year OS rate (73%) than CCL4^low^CCL20^low^ (40.9%) or CCL4^low^CCL20^high^ patients (50%). Recently, nomogram-based immunoprofile, a comprehensive scoring system including TNM stage, PD-L1 expression, and infiltration of CD8^+^/Foxp3^+^/CD33^+^ cells, has been developed for prognosis prediction in EC patients [78]. Based on the C-index calculation and receiver operating characteristic (ROC) analysis, this scoring system was able to separate same-stage patients into different risk subgroups, showing superior accuracy for survival prediction compared with TNM staging system.

## Immunotherapy strategies for EC

Strategies for improving the antitumor immunity in EC could be designed to increase the initiation of the tumor-killing immune response or to rescue the existing antitumor immune response that is suppressed in tumors. Vaccination therapy and adoptive T-cell therapy could be used to endow patients with an extra tumor-killing immune response, and immune checkpoint blockade could be used to normalize autogenous antitumor immunity in tumors.

### Tumor vaccination

As described above, TAAs and specific neoantigens with high immunogenicity have been identified in EC. These antigens can be utilized to design peptide vaccines for immunotherapy in EC. Among the TAAs, peptide vaccines based on TTK and NY-ESO-1 have been investigated in clinical trials for ESCC. Clinical trials using a vaccine made up of multiple peptides, including TTK, lymphocyte antigen-6 complex locus K (LY6K), and insulin-like growth factor-II mRNA binding protein-3 (IMP3), showed that vaccination with multiple peptides was able to induce HLA-A*2402-dependent clinical responses in ESCC patients [79, 80]. Another multiple-peptide vaccination strategy using TTK, up-regulated lung cancer 10 (URLC10), kinase of the outer chloroplast membrane 1 (KOC1), vascular endothelial growth factor receptor 1 (VEGFR1), and vascular endothelial growth factor receptor 2 (VEGFR2) also showed promising results in a phase I clinical trial of ESCC with chemoradiotherapy [81]. These studies warrant further clinical investigations of TTK-based multiple-peptide vaccines. One phase I clinical trial in patients with advanced ESCC demonstrated the safety and immunogenicity of a vaccination strategy based on NY-ESO-1: a combination of cholesterol-containing hydrophobic amylopectin and NY-ESO-1 protein (CHPNY-ESO-1) [82]. However, further studies are required to investigate the clinical benefit of NY-ESO-1-based vaccination in the future. Neoantigen-targeted cancer vaccines have shown antitumor efficacy on ESCC in recent preclinical studies [83]. To the best of our knowledge, clinical trials of neoantigen-targeted cancer vaccines in EC patients have not been reported.

### Adoptive T-cell therapy

TAAs and specific neoantigens can also serve as targets for adoptive T-cell therapy using chimeric antigen receptor (CAR)-T cells or antigen-specific T cell receptor (TCR) transgenic T cells. Adoptive T-cell therapy also includes locoregional immunotherapy with ex vivo-generated T cells stimulated by autologous tumor cells. Clinical trials of adoptive T-cell therapy with autologous tumor cell-stimulated cytotoxic T lymphocytes (AuTLs) in EC patients have been arranged since 2000 [84, 85]. AuTLs showed tolerable toxicity and clinical benefits when used to treat advanced and recurrent EC. In a clinical trial of advanced EC, one of 11 patients receiving locoregional administration of AuTLs had complete response, and three had partial response, while half of these patients had progressive disease [84]. Further studies intending to reduce the toxicity and enhance the efficiency of this strategy are required in the future. Recently, more researches have focused on the application of CAR-T cells or TCR T cells in the adoptive cellular immunotherapy of EC. Kageyama et al. [86] conducted a first-in-man clinical trial of TCR T cell transfer in patients with recurrent MAGE-A4-positive EC and showed that seven of ten patients who received adoptive transfer of MAGE-A4 T cell receptor gene-transduced lymphocytes had tumor progression within 2 months, while three patients with minimal tumors survived more than 27 months post treatment. To improve the treatment outcome of TCR T cell therapy, preclinical and clinical studies are being conducted to investigate novel TCR T cell therapy strategies, including neoantigen-targeting TCR T cell therapy in EC [17, 87]. Additionally, CAR-T cells targeting EphA2 have been used to treat EC and have shown antitumor effects in preclinical studies [88]. However, no clinical trial of CAR-T cell therapy in EC patients has been reported. TIL, an important candidate of adoptive T cell therapy, exhibited antitumor activities in preclinical or clinical studies on cancer therapy of several solid tumors, including melanoma and ovarian cancer [89, 90]. In EC, TIL levels were significantly correlated with prolonged patient survival [75, 91]. TILs have also been used for the isolation of autologous tumor-specific T cell receptor and the construction of TCR T cells [87]. However, to the best of our knowledge, studies using adoptive TIL transfer for the treatment of EC have not been reported.

### Inhibition of immune checkpoints

Except for active and passive immunization, enhanced antitumor immunity can also be obtained by rescuing the existing tumor-killing immune response, which is suppressed by TME-derived regulators, including immune checkpoints. Several clinical trials have investigated the safety and efficacy of anti-PD-1 antibodies, including pembrolizumab and nivolumab, on EC. As can be seen in Table 2, pembrolizumab has been used in different phases of clinical trials as a second- or later-line therapy for EC and has shown safety and clinically meaningful effects in both ESCCs and EACs [8]. Nivolumab also showed promising safety and antitumor activity in patients with ESCC in a clinical trial in Japan [8]. Clinical trials using other PD-L1/PD-1 inhibitors, such as SHR-1210, for EC therapy are also ongoing [18]. As described above, other immune checkpoints, including CTLA-4, are also commonly found in EC. A clinical trial has demonstrated the safety and efficacy of a combined therapy with anti-CTLA-4 antibodies (ipilimumab) and anti-PD-1 antibodies (nivolumab) in advanced EC (Table 2), showing that the outcome of patients receiving combined therapy was better than that of those receiving nivolumab monotherapy [92]. However, the adverse events in patients treated with CTLA4 inhibitors were more common and more serious than those with PD-1 inhibitors [93]. Therefore, the development of effective strategies to reduce the adverse events of CTLA-4 inhibitors is required for immunotherapy targeting CTLA-4 in EC.

**Table 2:** Clinical trials of immune checkpoint blockade in EC

Target	Drug	Treatment	Phase	Study ID	Outcome summary
PD-1	Pembrolizumab	Pembrolizumab alone	IB	Keynote-028	ORR 30% in PD-L1^+^ EC
		Pembrolizumab alone	II	Keynote-180 (NCT02559687)	ORR 14.3% in ESCC and 5.2% in EAC
		Pembrolizumab vs. irinotecan or taxanes	III	Keynote-181 (NCT02564263)	Median OS in ESCCs: 8.2 vs. 7.1 months ORR in ESCCs: 16.7% vs. 7.4%
		Pembrolizumab + cisplatin and 5-fluorouracil vs. placebo	III	Keynote-590 (NCT03189719)	Ongoing
PD-1	Nivolumab	Nivolumab vs. taxanes	III	NCT02569242	Median OS in ESCCs: 10.9 *vs.* 8.4 months
		Nivolumab alone	II	JapicCTI-142422	17% of ESCC patients had a centrally assessed objective response
		Nivolumab vs. placebo	III	Checkmate-577 (NCT02743494)	Ongoing
		Nivolumab + ipilimumab or nivolumab + fluorouracil + cisplatin vs. fluorouracil + cisplatin	III	Checkmate-648 (NCT03143153)	Ongoing
	SHR-1210	SHR-1210 alone	I	NCT0274293	ORR 30% and median PFS 3.6 months in ESCC
		SHR-1210 vs. docetaxel or irinotecan	III	NCT03099382	N/A
CTLA-4	Ipilimumab	N/A	I	NCT01738139	Ongoing
PD-1/CTLA-4	Nivolumab/ipilimumab	Nivolumab (3 mg/kg) vs. nivolumab (1 mg/kg) + ipilimumab (3 mg/kg) vs. nivolumab (3 mg/kg) + ipilimumab (1 mg/kg)	I/II	CheckMate-032	ORR in patients with gastric, esophageal, or gastroesophageal junction cancer: 12% vs. 24% vs. 8%

*N/A* not available, *ORR* objective response rate, *EC* esophageal cancer, *ESCC* esophageal squamous cell carcinoma, *EAC* esophageal adenocarcinoma, *OS* overall survival, *PFS* progression-free survival, *PD-1* programmed death protein-1, *CTLA-4* cytotoxic T lymphocyte-associated protein 4

In addition to normalizing the tumor-killing activities of T cells by blocking PD-L1/PD-1 signaling or B7/CTLA-4 signaling, the strategies to rescue the existing antitumor immune response that is suppressed in cancers should also include the blockade of immune checkpoints that inhibit the functions of APCs, since the presentation of tumor antigens by APCs is critical for T cell cross-priming. CD47 expressed on tumor cells interacts with the receptors on M1-like TAMs and thus impairs their phagocytic activity [94]. Therefore, CD47-blocking strategies could restore the engulfing and tumor-antigen-presenting activities of M1-like TAMs and thus enhance the priming of effective CD8^+^ T cells. Anti-CD47 therapy with a CD47 antagonist increased the tumor infiltration of CD8^+^ T cells in a preclinical model of ESCC. Moreover, anti-CD47 treatment enhanced the efficacy of anti-PD-1 and CTLA-4 therapy in ESCC [95]. Notably, in this study, the effects of anti-CD47 therapy depended on the function of DCs, suggesting that CD47 overexpression may impair the function of not only M1-like TAMs but also DCs. Although there are clinical trials of anti-CD47 therapy ongoing in other solid tumors [94], its use has not been reported in EC.

## Future prospects

**Fig. 1:**
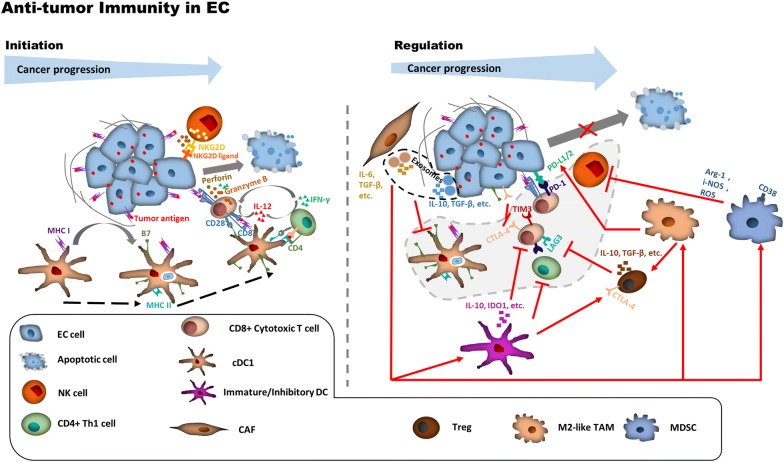
The initiation and regulation of antitumor immunity in EC. EC cells harbor abundant tumor antigens and are able to induce antitumor immune response, particularly in the early stage of EC. However, during tumor development, EC cells acquire the ability to escape immune surveillance through various ways. *EC* esophageal cancer, *NKG2D* natural killer group 2D, *IL-12* interleukin-12, *IFN-γ* interferon-γ, *MHC-I* major histocompatibility complex class I, *MHC-II* major histocompatibility complex class II, *IL-6* interleukin-6, *TGF-β* transforming growth factor-β, *IL-10* interleukin-10, *PD-L1/2* programmed death-ligand 1/2, *PD-1* programmed cell death protein 1, *TIM-3* T-cell immunoglobulin and mucin-domain containing-3, *CTLA-4* cytotoxic T lymphocyte-associated protein 4, *LAG-3*, lymphocyte-activation gene 3, *IDO1* indoleamine 2,3-dioxygenase 1, *ROS* reactive oxygen species, *i-NOS* inducible nitric oxide synthase, *Arg-1* Arginase-1, *NK* natural killer, *Th1* type 1 T helper, *cDC1* conventional type 1 dendritic cell, *CAF* cancer-associated fibroblast, *Treg* regulatory T cell, *TAM* tumor-associated macrophage, *MDSC* myeloid-derived suppressor cell

The immune landscape of EC is characterized by various TME-derived factors for the initiation of the tumor-killing immune response and the negative regulation of antitumor immunity (Fig. 1). The functions and mechanisms of some TME-derived factors for initiating or regulating antitumor immunity remain unclear, and further studies on them are required in the future. Strategies based on the immune landscape can be developed for prognosis and therapy in EC patients. Traditional prognosis prediction strategies for cancer are facing multiple challenges [31], and strategies using factors involved in the initiation or regulation of antitumor immunity may open a new window for cancer prognosis. Some factors of the TME immune landscape in EC are associated with cancer development, patient survival, or treatment response (Table 1), and the prognosis prediction strategies considering the combination of different immune checkpoints or different types of immune cells improved the accuracy of TME-derived factors in prediction of EC patient outcomes. However, more effective strategies by combining TME-derived factors (including both immune-activated and immune-suppressed factors) and pathologic criteria may be required for EC prognosis prediction in the future [31, 35, 43, 96]. A recent nomogram-based immunoprofile, including PD-L1 expression, infiltration of different types of immune cells, and TNM stage, has shown promising prospect in prognosis prediction using immune landscape in EC [78]. Moreover, immunoscore, a scoring system based on the quantification of cytotoxic and memory T cells in the core of tumor and in the tumor’s invasion margin has been shown as one of the strongest prognostic factors for colorectal cancer [97]. A similar scoring system could also be developed for accurate prognostic prediction of EC patients. Additionally, Tregs and MDSCs may have heterogenic phenotypes and diverse functions, and the consideration of diverse functions of these TME factors is required for the improvement of their prognostic value in EC. Notably, CD80 and CD86, which are expressed on both tumor cells and TILs, can interact with CD28 on T cells to activate costimulatory signaling and with CTLA-4 on activated T cells to initiate inhibitory signaling. The expression of CD80 and CD86 on tumor cells or TIICs (especially APCs) may have a bias to suppress or activate the antitumor immune response, respectively [98]. Therefore, distinguishing CD80 and CD86 expression between tumor cells and APCs is required for a more accurate prognosis prediction in EC patients. Similar to B7, PD-L1/2 can also be expressed on both tumor cells and TILs. Hatogai et al. [29] have shown that the combined levels of PD-L1 expression in both tumor cells and TILs have enhanced the accuracy of prognostic prediction compared with the level in each cell. The following improvements are required for the prognosis prediction of EC using PD-L1: (i) comprehensive evaluation of PD-L1 levels in both tumor cells and TILs and (ii) unified and standard principles for PD-L1 measurement.

Cancer immunotherapy strategies, including tumor vaccination, adoptive T cell therapy, and immune checkpoint blockade, have shown antitumor bio-activities in preclinical and clinical studies of EC recently. However, most of them were based on single immunotherapy [8, 18, 79–82, 84] and would be difficult to efficiently suppress cancer progression for a long time due to the intricate crosstalk regulation of immune evasion in EC. Therefore, combined immunotherapy strategies are also required for EC. The combination of PD-1 inhibition and CTLA-4 blockade [92] or of peptide vaccinations and TCR T cell therapy [86] has been reported in clinical trials, showing abilities to suppress tumor development in EC patients. IDO inhibitors [74] or CD47 antagonists [95] have the ability to restore the functions of APCs in antigen presentation and T cell priming. Such anti-IDO or anti-CD47 therapy can be used to enhance the efficacy of PD-1 inhibition. However, reducing the side effects of IDO inhibitors or CD47 antagonists needs to be considered. Tumor vaccination can also be used to enhance the generation of effective tumor-specific CD8^+^ T cells in individuals and thus improve the efficacy of PD-1 blockade. To improve the tumor-killing response to TCR T cell therapy, strategies for enhancing tumor infiltration of T cells are required. Since tumor-promoting CAFs have the ability to promote the synthesis of extracellular matrix [99] and the depletion of CAFs increases intratumoral drug uptake [100], CAF-targeting strategies could be developed to enhance the tumor infiltration of TCR T cells or CAR T cells in EC. Additionally, strategies for accurately predicting the treatment response are required to improve the efficacy of immunotherapy on EC. PD-L1 expression is commonly used to predict the therapeutic response of PD-1 inhibition in EC, and combined prognosis prediction strategies based on the PD-L1 expression and the presence or absence of TILs have also been established. However, these strategies remain ineffective for the accurate prognostic prediction of EC patients with PD-L1/PD-1-blocking therapy [5]. It’s worth noting that the responses to PD-L1/PD-1 immune checkpoint blockade could be observed in PD-L1-negative tumors [6, 101]. The antitumor efficacy of PD-L1/PD-1 inhibitors on PD-L1-negative tumors may be caused by the blockade of PD-L2/PD-1 signaling [102] or by the activation of tumor-killing NK cells [103]. A more comprehensive strategy is needed for the direction of immunotherapy with anti-PD-1 antibodies in EC, and a strategy using a combination of PD-L1 expression, MANA count, and TMB could be a potential choice [18]. High MSI and TMB could lead to high levels of tumor neoantigens, TILs, and immune checkpoints. High MSI is rarely found in EC [5], but high TMB and neoantigens are widely found in EC [9, 17, 18]. TMB serves as an independent predictor of immunotherapy response across multiple cancer types [104, 105]. The quantity of neoantigen was identified as a biomarker of immunogenic tumors, associated with patient survival, and able to predict response to immune checkpoint blockade [106, 107]. Therefore, TMB and neoantigen account can be used to design novel strategies for more accurate prediction of immunotherapy response. Different prognosis prediction strategies may be required for the direction of different types of immunotherapy for cancer patients. NY-ESO-1 expression could be used to predict the response to vaccination therapy in EC patients [24, 64]. More strategies for accurately predicting the response of different immunotherapies, including immune checkpoint inhibition, tumor vaccination, and adoptive T cell therapy, are required in the future. Additionally, different strategies for cancer prognosis prediction or therapy should be developed for ESCC and EAC, since the immune landscapes in ESCC and EAC could be different [15, 16, 26].

## Conclusions

Here, we discuss the immune landscape in EC, providing a clear picture for the initiation or regulation of the antitumor immune response in EC. This picture provides a theoretical basis for further studies of the regulation of antitumor immunity in EC. We also describe the strategies based on these immune landscapes for cancer prognostic prediction or therapy in EC and discuss the current bottlenecks and potential improvements for these strategies, thus providing directions to design novel and effective strategies for cancer prognostic prediction or therapy in EC.

## Data Availability

Not applicable.

## Abbreviations

EC: esophageal cancer
ESCC: esophageal squamous cell carcinoma
EAC: esophageal adenocarcinoma
CPS: combined positive score
TPS: tumor proportion score
PD-L1: programmed death-ligand 1
PD-1: programmed cell death protein 1
FDA: Food and Drug Administration
TME: tumor microenvironment
TAA: tumor-associated antigen
CTA: cancer/testis antigen
MAGE-A: melanoma-associated antigen-A
NY-ESO-1: New York esophageal squamous cell carcinoma 1
TTK: TTK protein kinase
PBMC: peripheral blood mononuclear cell
CTL: cytotoxic T lymphocyte
APC: antigen-presenting cell
DC: dendritic cell
TIL: tumor-infiltrating lymphocyte
MHC-I: major histocompatibility complex class 1
HLA-2: human leukocyte antigen 2
Th1: type 1 T helper
NK: natural killer
CTLA-4: cytotoxic T lymphocyte-associated protein 4
TIM-3: T-cell immunoglobulin and mucin-domain containing-3
LAG-3: lymphocyte-activation gene 3
TIIC: tumor-infiltrating immune cell
IDO1: indoleamine 2,3-dioxygenase 1
TGF-β: transforming growth factor-β
TAM: tumor-associated macrophage
MDSC: myeloid-derived suppressor cell
Treg: regulatory T cell
IL-10: interleukin 10
IL-6: interleukin 6
IL-8: interleukin 8
CAF: cancer-associated fibroblast
VEGF: vascular endothelial growth factor
CCL2: C–C motif chemokine ligand 2
CCL5: C–C motif ligand 5
CCL20: C–C motif ligand 20
CCL17: C–C motif ligand 17
CCL22: C–C motif ligand 22
ALDH1: aldehyde dehydrogenase 1
IL-17A: interleukin-17A
PDAC: pancreatic ductal adenocarcinomas
FAP: fibroblast activation protein
MSI: microsatellite instability
MANA: mutation-associated neoantigen
OS: overall survival
IFN-γ: interferon-γ
GramB: granzyme B
pCR: pathologic complete response
ROC: receiver operating characteristic
LY6 K: lymphocyte antigen-6 complex locus K
IMP3: insulin-like growth factor-II mRNA binding protein-3
URLC10: upregulated lung cancer 10
KOC1: kinase of the outer chloroplast membrane 1
VEGFR1: vascular endothelial growth factor receptor 1
VEGFR2: vascular endothelial growth factor receptor 2
CAR: chimeric antigen receptor
TCR: T cell receptor
AuTL: autologous tumor-cell-stimulated cytotoxic T lymphocyte
TMB: tumor mutational burden
NKG2D: natural killer group 2D
i-NOS: inducible nitric oxide synthase
cDC1: conventional type 1 dendritic cell
Arg-1: Arginase-1
